# Colloidal Gold-Mediated Delivery of Bleomycin for Improved Outcome in Chemotherapy

**DOI:** 10.3390/nano6030048

**Published:** 2016-03-10

**Authors:** Celina Yang, Jamie Uertz, Devika B. Chithrani

**Affiliations:** 1 Department of Physics, Ryerson University, Toronto, ON M5B 2K3, Canada; celina.yang@ryerson.ca; 2 CytoViva Inc., 570 Devall Drive, Auburn, AL 36832, USA; jamie.uertz@cytoviva.com; 3 Keenan Research Centre, Li Ka Shing Knowledge Institute, St. Michael’s Hospital, Toronto, ON M5B 1W8, Canada

**Keywords:** colloidal gold nanoparticles, anticancer drug, bleomycin, DNA double strand breaks, nanomedicine, drug delivery

## Abstract

Nanoparticles (NPs) can be used to overcome the side effects of poor distribution of anticancer drugs. Among other NPs, colloidal gold nanoparticles (GNPs) offer the possibility of transporting major quantities of drugs due to their large surface-to-volume ratio. This is while confining these anticancer drugs as closely as possible to their biological targets through passive and active targeting, thus ensuring limited harmful systemic distribution. In this study, we chose to use bleomycin (BLM) as the anticancer drug due to its limited therapeutic efficiency (harmful side effects). BLM was conjugated onto GNPs through a thiol bond. The effectiveness of the chemotherapeutic drug, BLM, is observed by visualizing DNA double strand breaks and by calculating the survival fraction. The action of the drug (where the drug takes effect) is known to be in the nucleus, and our experiments have shown that some of the GNPs carrying BLM were present in the nucleus. The use of GNPs to deliver BLM increased the delivery and therapeutic efficacy of the drug. Having a better control over delivery of anticancer drugs using GNPs will establish a more successful NP-based platform for a combined therapeutic approach. This is due to the fact that GNPs can also be used as radiation dose enhancers in cancer research.

## 1. Introduction

Side effects of anticancer drugs still remain a problem in cancer treatment [1]. This is partially due to poor distribution of anticancer agents. The side effects may be reduced by improving the bioavailability of the drug in the tumor region [2,3,4,5]. Hence, nanoparticle (NP)-based drug delivery systems have been implemented by several research groups, as these drug delivery systems can provide improvements to the free drug by increasing *in vivo* stability and biodistribution [6,7,8,9,10,11,12,13,14]. Among other NP systems, gold nanoparticles (GNPs) are an ideal drug-delivery scaffold because they are known to be nontoxic and nonimmunogenic [15,16,17]. The chemical and physical properties of GNPs are beneficial in transporting small molecules, including anticancer drugs. The size of GNPs can be tailored to take advantage of passive targeting into unhealthy tissues and enhanced permeation and retention (EPR) effects (due to gaps in leaky tumor blood vessels) [14]. Enhanced selectivity can be further achieved by targeting overexpressed receptors in cancer cells [9]. The GNP core is inert and non-toxic, while functionalization can be easily performed through thiol linkages [15]. In addition, GNPs being able to interact with thiols can establish a basis for providing an effective way of intracellular release [14]. GNPs are being used as radiation dose enhancers in radiation therapy. The ultimate goal of GNP-based platforms will be the implementation of a combined treatment of radiation therapy and chemotherapy to tumors while causing minimum side effects. Many of the side effects of anticancer drugs are caused because of their nonspecific attack on all rapidly dividing cells. Hence, GNPs can be used to resolve certain limitations in chemotherapy such as side effects through targeting and effective loading [18].

One of the most startling results of GNP uptake studies was the difference in size-dependence of GNP uptake at monolayer and multilayer (tissue-like) levels [19,20]. At the monolayer level, GNPs of diameter 50 nm have a higher cellular uptake as opposed to smaller or larger GNPs [19,21,22,23,24,25]. This phenomenon is largely explained by the energy interplay between the receptor-ligand binding process and the deformation energy of the cell [21,26]. At the multilayer (tissue-like) level, smaller NPs penetrated better resulting in a higher cell uptake [27,28,29,30,31,32,33]. These results suggest the possibility that the extra cellular matrix (ECM) may play a large role in determining the transport dynamics of GNPs in tissue-like structures. Given that transport through ECM is diffusion-dependent, there exists, at the very least, a competing dynamic that predicts GNP transport to be inversely proportional to GNP size. Once NPs leave the tumor blood vessels, it is necessary for them to penetrate through the tumor tissue to undergo uptake by individual cells. The therapeutic response can be improved if we can target these GNPs into individual cells in the core of tumor tissue. Based on the previous work at tissue level, smaller NPs are preferred as compared to larger NPs [27,28,29,30,31,32,33]. In addition, if properly functionalized, GNPs of sizes smaller than 25 nm can be transported into the nucleus [34]. Nuclear targeted GNPs have shown higher radiation dose enhancement in radiation therapy [35]. If we can direct some of the drug conjugated GNPs into the nucleus without having to use a cocktail of peptides, it will open the door for us to use combined therapy (radiation therapy plus chemotherapy) to overcome the therapeutic resistance of cancer cells.

In this study, we used smaller NPs for two main reasons: (a) smaller NPs will penetrate better in tumor tissue once they leave the tumor blood vessels; and (b) the improved delivery of drugs since the surface-to-volume ratio is higher for smaller NPs. We had two goals: one was to investigate whether the therapeutic efficacy of the drug changes once it is conjugated onto the GNP surface; and the second was to investigate whether NPs can reach the nucleus since the action of the drug is mainly in the nucleus. We used bleomycin (BLM) as the anticancer drug for our study. This drug could be easily conjugated onto the GNP surface using a thiol bond. BLM is one of the most potent natural anticancer drugs available and has been used for chemotherapeutic agents in clinical treatments of certain cancers [36,37]. However, the therapeutic effectiveness of BLM is limited due to its side effects, most notably pulmonary toxicity [2]. The usage of this particular anticancer drug could be expanded if lower dosages could be delivered closer to the target and could be contained. It is also shown that it is necessary for BLM to bind to Deoxyribonucleic Acid (DNA) in order to cause the DNA double strand breaks (DSBs). DSBs are considered to be the most harmful of the DNA lesions that impair DNA integrity and genomic stability [38]. However, it is not fully known yet whether conjugating BLM onto the GNP surface (is referred to as GNP-BLM) will affect the efficacy of the drug or the pathway of the GNPs within the cells. Hence, in this study, we will try to answer the following questions:

1 Will the efficacy of the drug be compromised by conjugating them onto the GNP surface?
2 The action of the drug is through binding onto DNA. Does this mean NPs can also reach the nucleus?
3 Will drug conjugated GNPs (GNP-BLM) be more effective than the BLM alone (free drug)?

## 2. Results

### 2.1. Characterization of Nanoparticles

**Figure 1:**
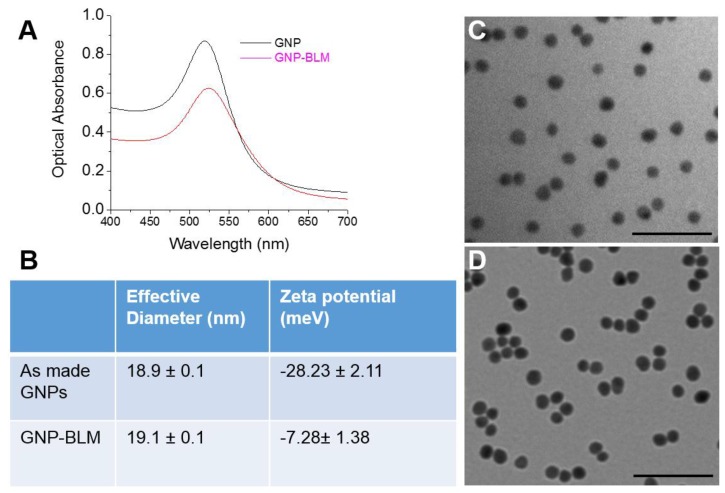
Characterization of gold nanoparticle-bleomycin (GNP-BLM) *vs.* as made (citrate-capped) GNPs. (**A**) Ultraviolet-visible absorption spectra of as made GNP and GNP-BLM; (**B**) Hydrodynamic diameter and zeta-potential of as made GNPs and GNP-BLM; (**C**,**D**) Transmission electron microscopy (TEM) images of as made GNPs and GNP-BLM, respectively. The scale bar is 100 nm.

The Ultraviolet-Visible (UV-Vis) spectra of citrate capped GNP had a peak of 517 nm (Figure 1A) which corresponds to approximately ~10 nm in core diameter [39,40]. The GNP surface was modified with the penta-peptide (peptide sequence: CALNN) and a peptide containing RGD binding domain (peptide sequence: CKKKKKKGG**RGD**MFG) in addition to BLM. The penta-peptide was used to stabilize the NP for conjugation with BLM and the RGD peptide. The RGD peptide was used to increase the NP uptake as discussed in the next section. Now onwards, GNPs conjugated with BLM along with the above mentioned two peptides will be referred to as GNP-BLM. The free drug, bleomycin, is referred to as BLM. The peak was red shifted to 519 nm for the GNP-BLM complex due to the presence of BLM and peptides. The shape of the spectrum remains the same up to 48 h post conjugation, which signifies that aggregation of the complex does not occur during this time period. The hydrodynamic radius was also measured using the dynamic light scattering technique (see Figure 1B). Adding BLM increased the hydrodynamic diameter by less than 1 nm. This corresponds to the approximate size of BLM [41]. TEM images of as-made GNPs and GNP-BLMs are shown in Figure 1C,D, respectively. According to TEM images, a thin coating around the NPs can be seen for GNP-BLM. The stabilizing of the GNP surface with a penta-peptide followed by the addition of an RGD peptide and BLM was monitored using the above mentioned characterization techniques. Additional characterization information is given in the Supplementary Material, Section S1.

### 2.2. Mapping of NP Distribution Using Hyperspectral Imaging Technique

**Figure 2:**
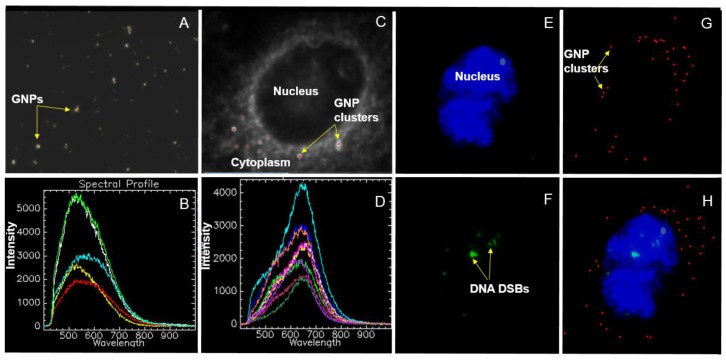
Imaging of GNPs using hyperspectral imaging technique: (**A**) Dark-field image of GNPs; (**B**) reflectance spectra of a few GNPs shown in (**A**); (**C**) dark-field image of an unstained cell with internalized GNP clusters (bright white spots); and (**D**) reflectance spectra of few GNP clusters localized within the cell shown in (**C**). (**E–H**) Hyperspectral image of a stained (nucleus and DNA Double Strand Breaks (DSBs)) cell. Three different channels were used to display nucleus, GNPs, and DNA DSBs. Blue areas are nuclei of the cell (**E**); green dots represent DNA DSBs (**F**); and red dots represent GNP clusters localized within cells (**G**); (**H**) A merged image of the three channels corresponding to the nucleus, GNPs, and DNA DSBs).

The hyperspectral imaging (HSI) technology is a novel technique that can be used not only for optical observation but also for spectral confirmation of NPs. Figure 2A is a dark-field image of GNPs of size 10 nm. The small white bright dots like structures are GNP clusters. The imaging of GNPs without any optical probes on them is possible due to their higher scattering cross section of visible light. Hence, it was not necessary to optically label them. The HSI technique was used to obtain reflectance spectra corresponding to each pixel in the dark-field image. Figure 2B shows some of the reflectance spectra taken from few GNPs shown in Figure 2A. Figure 2C is an HSI image of a cross section of an unstained cell across the nucleus. GNP clusters localized within the cells appear as bright dots. No bright dots are seen within the nucleus since as-made GNPs cannot reach the nucleus. The spectral profile taken from the cytoplasm and nucleus was fairly flat in contrast to the ones from GNPs localized within the cell (see Supplementary Material, Section S2 for more details). It is possible to map the image plane chosen using a reference reflectance spectra of GNPs. Figure 2C represent such an image where where red dots represent matching GNP spectra to the reference spectrum of GNPs. Figure 2D represents individual reflectance spectra from a few GNP clusters within the cell. These results confirm that the bright dots observed through HSI microscopy images are GNPs and not artifacts. For this particular study, it was important to visualize the nucleus and DNA DSBs in addition to GNPs in order to investigate the co-localization of GNPs and DNA DSBs within the nucleus. We used optical labels for the nucleus and DNA DSBs, as discussed in the Experimental Section. Figure 2E–H show the three different channels along with the merged image: blue areas are nuclei of cells (Figure 2E), Green dots represent DNA DSBs (Figure 2F), red dots represent NP clusters localized within cells (Figure 2G), and a merged image (Figure 2H).

### 2.3. Cellular Uptake of BLM Conjugated GNPs

**Figure 3:**
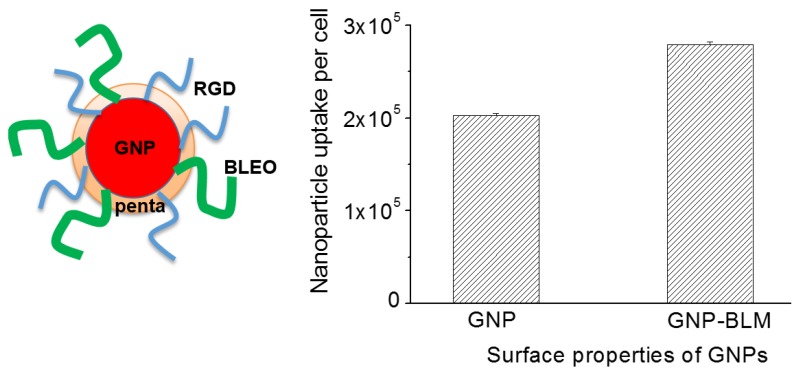
Cellular uptake of GNP-BLM *vs.* as-made GNPs. (**Left**) Schematic showing different molecules on the GNP-BLM complex. (**Right**) Quantified uptake of GNP-BLM *vs.* as-made GNPs using the Inductively Coupled Plasmon Atomic Emission Spectroscopy (ICP-AES) technique (*n* = 3).

We first tested the viability of the cells in the presence of as-made (or citrate capped) GNPs *vs.* control (cells with no GNPs) using a clonogenic cell survival assay and DNA DSBs assay as illustrated in the Supplementary Material, Section S3. The viability of the cells was not compromised in the presence of as-made GNPs at the concentration used in this study. For this study, the GNP surface was modified with a penta-peptide, an RGD-peptide, and BLM. The NP complex is referred to as GNP-BLM for simplicity. A penta-peptide was used to stabilize as-made GNPs for BLM conjugation while an RGD-peptide was used to increase the NP uptake and also to influence NP release into the cytoplasm. The release of NPs into the cell cytoplasm in the presence of an RGD-peptide is illustrated in Supplementary Material, Section S4. Figure 3 shows that the uptake of GNP-BLM was ~35% higher than as-made GNPs and we speculate that it is due to the presence of an RGD-peptide. For example, uptake of as-made GNPs was increased by a factor of two after functionalizing them with RGD-peptide as illustrated in Supplementary Material, Section S1. Additional details related to variation of NP uptake as we modify the surface with peptides is given in Supplementary Material, Section S1. The increase in uptake of GNP-BLM could be due to their lower negative charge and the presence of an RGD-peptide containing integrin binding domain (RGD). Our previous cellular uptake data proved that the cells targeted with a peptide containing RGD components had the highest NP uptake as compared to cells targeted with as-made GNPs [34]. It is believed that the synthetic peptide with RGD domain supplemented an additional driving force for entry of NPs into cells with over expressed integrin binding domain [34]. In the next section, we will investigate the therapeutic efficacy of GNP-BLM *vs.* BLM (free drug).

### 2.4. Therapeutic Efficacy Due to GNP-BLM vs BLM

**Figure 4:**
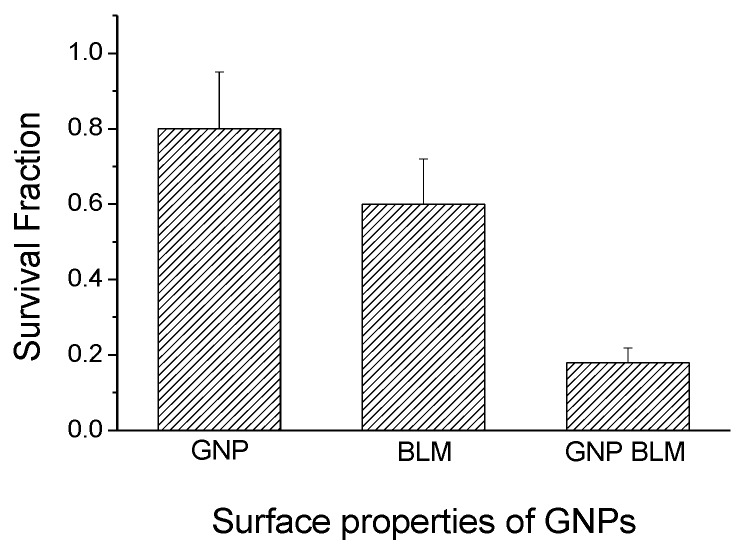
The long-term therapeutic efficacy of drug delivery using GNPs was measured using a clonogenic survival assay. The survival fraction of cells treated with GNP-BLM was lower compared to cells treated BLM alone (*n* = 3).

We used a clonogenic survival assay, a cell apoptotic assay, and a DNA DSBs assay to determine the therapeutic efficacy of BLM (free drug) *vs.* the GNP-BLM complex. The clonogenic assay measures the damage to cells over a period of two to three weeks while DNA DSBs and the apoptotic assay measures the damage to cell over a short period of time (24 h after the treatment). The survival fraction (SF) of cells was evaluated from clonogenic assay experiments (as shown in Figure 4). Survival fraction was lower when cells were treated with GNP-BLM *vs.* BLM (see Supplementary Material, Section S5 for additional information). This signifies that conjugating BLM onto the GNP surface improves delivery of the drug into the cell. Having an RGD-peptide in addition to a penta-peptide not only improved the internalization of a GNP-BLM complex but also enhanced the possibility of escaping the endo-lyso path into cytoplasm (see Supplementary Material, Section S4). This would have increased the residency time of NPs within the cell resulting in an enhanced release of BLM into the cell cytoplasm for nuclear delivery.

**Figure 5:**
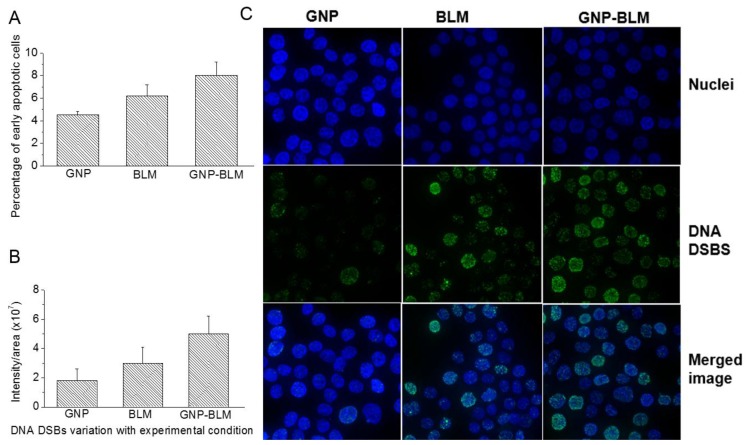
Measuring the efficacy of the drug quantitatively and qualitatively using a cell apoptotic assay and a DNA DSBs assay. (**A**) The average percentages of early apoptotic cells for the control, BLM-treated, and the GNP-BLM treated were 4.5%, 6.3%, and 7.8%, respectively; (**B**) Quantified DNA DSBs for cells treated with as-made GNPs, BLM alone, and the GNP-BLM complex; (**C**) Qualitative optical imaging data corresponding to DNA DSBs for cells treated with as-made GNPs (first column), BLM alone (second column), and the GNP-BLM complex (third column). The first row represents cells stained with DAPI (4',6-diamidino-2-phenylindole) for nuclei staining, the second row represents reflects DNA DSBs (p53-binding protein 1 (53BP1) protein staining with optically tagged antibodies), and the third row represents an overlay of channels corresponding to DNA DSBs and the nucleus.

Based on early apoptosis data, there were no significant differences in the percentages of late apoptotic cells for the different treatments (see Figure 5A). Several cellular events occur during apoptosis and one is the loss of cell membrane asymmetry, where the phosphatidyl serine (PS) is exposed to the external leaflet. The cell viability can be assessed with annexin V and propedium iodide (PI) staining assays. The annexin V protein conjugated onto a fluorophore stains for PS externalization. The membrane impermeable PI can probe for membrane integrity. In this study, there were no significant differences in the case of late apoptosis for after cell treatment with GNPs, BLM, and GNP-BLM complex. This signifies that the incubation of BLM or GNP-BLM complex does not compromise the membrane integrity within 24 h. The average percentages of early apoptotic cells for the control, bleomycin-treated, and the GNP-BLM treated were 4.5%, 6.3%, and 7.8%, respectively (see Figure 5A).

Figure 5B,C show the quantitative and qualitative results of the DNA DSBs assay (see Supplementary Material, Section S5 and S6 for additional information). DNA DSBs are considered to be the most harmful of the DNA lesions that impair DNA integrity and genomic stability [38]. It has been shown before that one such DNA DSB, which is not repaired, would be enough to cause a growth arrest in a cell causing cell death [38]. An important regulator of DSB signaling is a p53-binding protein 1 (53BP1). When DSBs are detected, 53BP1 rapidly accumulated on the chromatin surrounding the break site during the initial stages of the DNA damage response, driven by a signaling cascade [42]. When these proteins are labeled, immunofluorescently, it allows for the visualization of discrete foci and the density in the nucleus is proportional to the amount of unrepaired DNA DSBs in the cell [43]. Figure 5B shows that there were enhanced DNA DSBs when the GNP-BLM was used *vs.* BLM (see Supplementary Material, Section S6 for additional information).

## 3. Discussion

The polymer and lipid based NP drug delivery systems are the most studied drug delivery systems [44,45,46,47]. However, recent research shows that inorganic NPs, such as GNPs, as promising NP-platforms can be effectively used for improved anticancer drug delivery. GNPs are an ideal drug-delivery system since they are nontoxic and nonimmunogenic at clinically relevant concentrations [15,16]. In addition, GNPs are being used as radiation dose enhancers in radiation therapy. Hence, the ultimate goal of GNP-based platforms will be the implementation of a combined treatment of radiation therapy and chemotherapy to tumors while causing minimal side effects. Many of the side effects due to anticancer drugs is a result of their effect on all rapidly dividing healthy cells. Hence, GNPs can be used to resolve certain limitations in chemotherapy through controlled delivery of anticancer drugs specifically to cancer cells [18].

**Figure 6:**
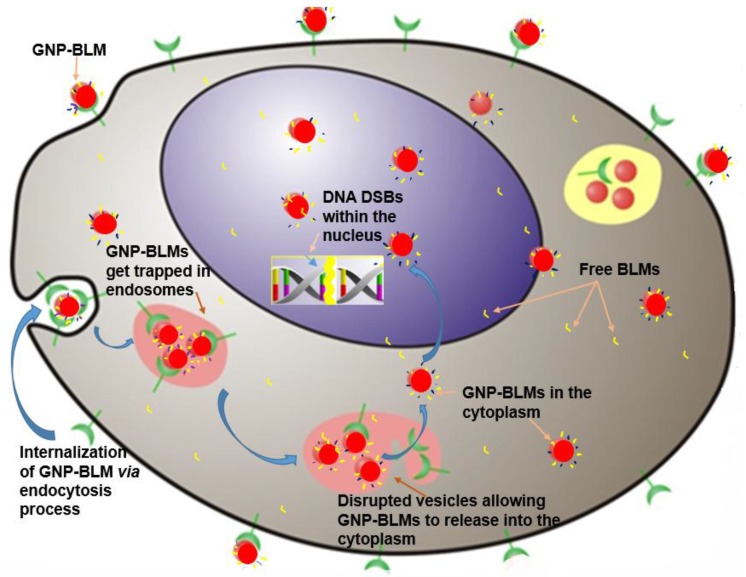
Schematic diagram explaining the pathway of the GNP-BLM complex. The GNP-BLM complex enters the cells via the regular endocytosis process and becomes trapped in the endo-lyso path. A peptide with an RGD domain on GNPs allows them to escape from endo-lyso path and enter the cytoplasm for nuclear delivery. BLM can also be released into the cytoplasm from GNP surface due to the changes in the physiological condition within the cell. Free BLM can also enter the nucleus as well. GNPs with BLM molecules still present on the surface could also reach the nucleus.

As shown in Figure 5 and Figure 6, it was possible to improve the delivery of the anticancer drug, BLM, into cancer cells by using GNPs. It is known that GNPs internalize the cells via the regular endocytosis process. NPs become trapped in endosomes and lysosomes before they are excreted through the cell. Hence, it is important for the GNP-BLM complex to release the BLM within these vesicles or in the cytoplasm for nuclear delivery. The release of the drugs from GNPs is in response to an intracellular biological signal of cancer cells. The change in pH in cellular compartments, such as endosomes and lysosomes compartments where NPs are trapped, presence of enzymes in lysosomes, and redox potential within the cytoplasm and the cell nucleus could lead to activation of intracellular signals that could lead to release of the drugs from NP surface [47]. However, it would be beneficial if anticancer drugs can be delivered and released into specific cellular compartments such as the nucleus using GNPs. GNPs are being used for radiation dose enhancement and GNPs localized within the nucleus showed higher dose enhancement. Since action of BLM is within the nucleus, we also wanted to investigate whether these NPs can reach the nucleus if some of the drug was still present on the NP surface. For this we needed to facilitate the escape of the endo-lyso path for GNPs. We used a RGD-peptide for this reason. To conclude, we used a RGD peptide along with BLM for the following reasons.

(1) A RGD peptide on the GNP-BLM complex allows for the release of these GNP-BLMs from these vesicles into the cytoplasm [34]. This will provide more time for the drug to be released from NPs into the cytoplasm.
(2) BLM is known to reach the nucleus and bind to DNA causing DNA DSBs. If a RGD peptide assists the transport of the GNP-BLM complex to the cytoplasm, it is also possible that we can even transport some GNPs into the nucleus with the help of BLM (if they are still available on the GNP surface). This would benefit GNP mediated combined therapeutic strategies of chemotherapy and radiation therapy in the near future.

It is also important to use smaller NPs if we were to use them for nuclear delivery since nuclear pores are ~25 nm in dimension [34]. It is also beneficial to use smaller NPs since they have a higher drug loading efficiency due to their higher surface to volume ratio. They also penetrate better in tumor tissue. However, one of the drawbacks of using smaller NPs is that there is lower uptake efficiency. We were able to enhance the uptake of smaller NPs by using a peptide containing RGD domain, as illustrated in Figure 3. The RGD-peptide added a driving force for interaction between the GNP-BLM and integrin binding domain receptors (RGD) over expressed in the MDA MB231 cell line used in this study. Hence, we were able to improve the delivery of the drug into the cells using the GNP-BLM complex. Now, the question is whether the drugs delivered using GNPs are as efficient as BLM alone (or the free drug).

**Figure 7:**
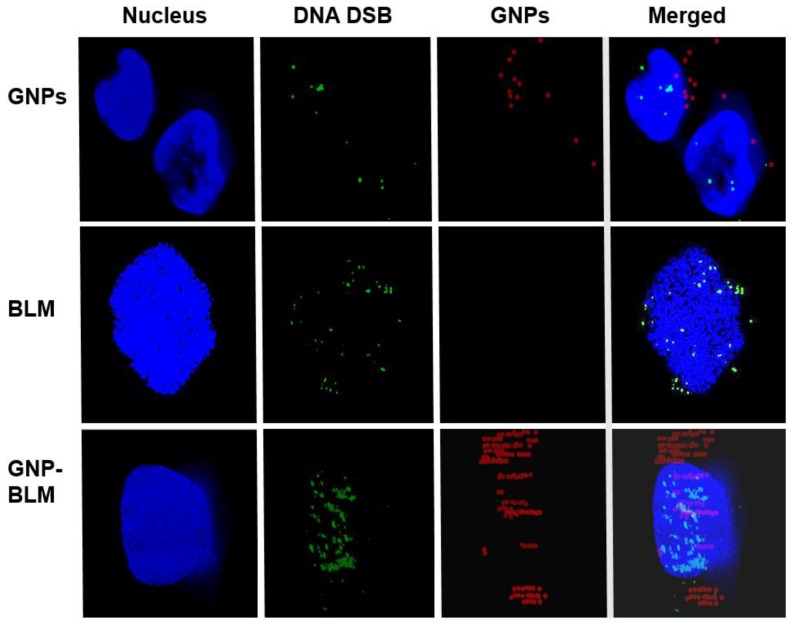
Hyperspectral mapping of GNP distribution and DNA DSBs within cells. Citrate capped GNPs did not enter the nucleus and caused less DNA DSBs (**top** panel). Free drugs caused more DNA DSBs (**middle** panel). The GNP-BLM complex entered the nucleus and caused more DNA DSBs (**Bottom** panel).

We completed three assays to verify the effectiveness of the drug delivered using GNPs (GNP-BLM) *vs.* free BLM as illustrated in Figure 4 and Figure 5. A clonogenic survival assay, an early apoptotic assay, and a DNA DSBs assay showed an enhanced therapeutic effect when cells were treated with GNP-BLM *vs.* free BLM. Figure 7 further illustrates that there was an enhancement in DNA DSBs when cells were treated with GNP-BLM (bottom panel) *vs.* free BLM (middle panel). Previously, Jain *et al.* treated MDA-MB-231 cells with 12 μM 1.9 nm GNPs and different concentrations of BLM in the μg/mL range. It was shown that there were improved therapeutics when BLM was used with GNPs (GNP-BLM) *vs.* BLM alone [48]. However, it was not clear whether BLM was conjugated onto GNPs. In our study, we were able to reduce the concentration of BLM and GNPs by a factor of 1000. We were still able to see the improved therapeutics under such a low concentration of BLM due to enhanced delivery via GNPs. In addition, GNPs were also functionalized with a peptide containing RGD domain. An RGD-peptide has an affinity for integrin binning domain, RGD, on the cell membrane. This would result in enhanced uptake of GNP-BLMs (see Figure 3 and Figure 7). An RGD-peptide also assisted GNPs in escaping to the cytoplasm thus improving their residency time within the cell for releasing BLM into the cytoplasm for nuclear delivery or for allowing GNPs to reach the nucleus (if BLMs were present on the GNP surface).

**Figure 8:**
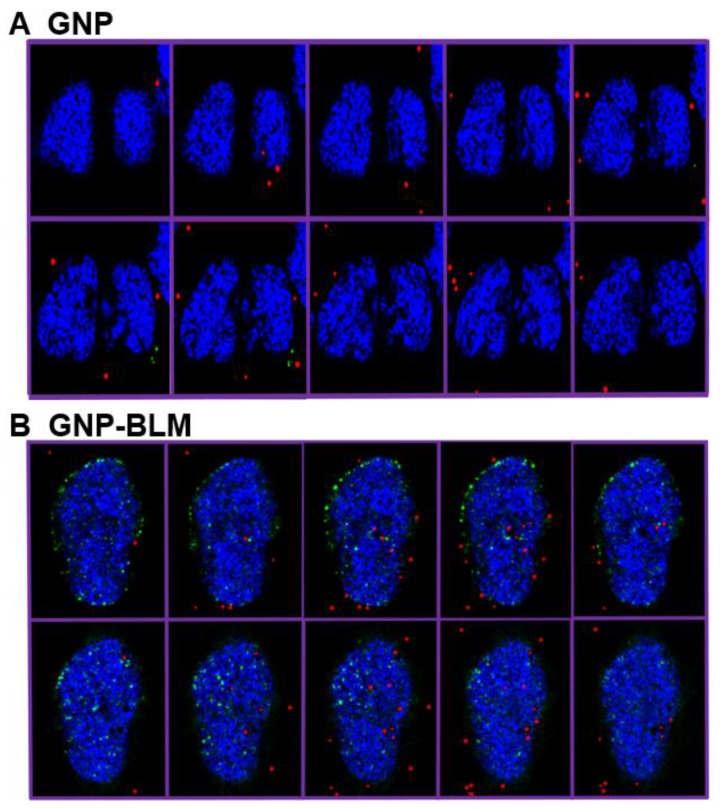
Hyperspectral mapping of GNP distribution and DNA DSBs in cells incubated with as-made GNPs (**A**) and GNP-BLM (**B**).(**A**) Different planes across the nuclei of the cells showed that there is localization of GNPs within the nucleus when they were conjugated with drug, bleomycin. (**B**) The cells incubated with citrate capped (as-made) GNPs could not reach the nucleus.

**Figure 9:**
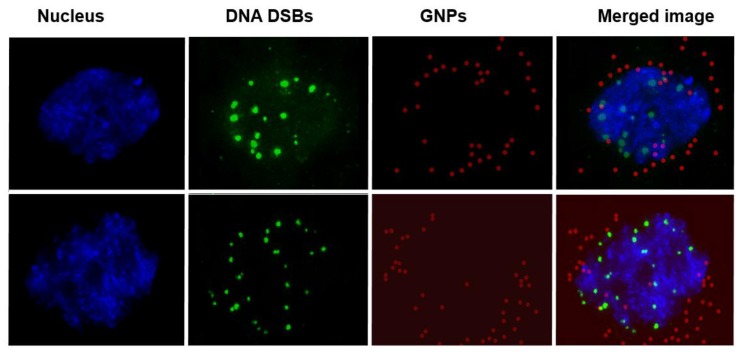
Hyperspectral mapping of GNP distribution and DNA DSBs in cells incubated with the GNP-BLM complex. More NPs were seen either in the nucleus or closer to the nuclear membrane.

Localization of the GNP-BLM within the nucleus was confirmed using the Hyper Spectral Imaging (HSI) technique, as shown in Figure 7. In this study, we imaged DNA DSBs in addition to GNPs. Hence we stained the nucleus with DAPI to further verify the co-localization of GNPs and DNA DSBs within the nucleus. In our optical images in Figure 7, the nucleus stained with DAPI appeared blue while GNPs and DNA DSBs appeared in red and green, respectively. Figure 7 (top panel) clearly shows that as-made GNPs were not localized within the nucleus, while some of the GNP-BLMs were localized within the nucleus (see Figure 7, bottom panel). The superposition of red and blue color produces a magenta color. In the bottom panel of Figure 7, the GNPs within the nucleus appear in magenta, thus confirming that they are localized within the nucleus. This is the first time that it was shown that GNPs were able to carry BLM to its biological target, DNA, without mediation of nuclear penetration molecules, such as peptides. Figure 8 further illustrates nuclear localization of GNP-BLMs. In Figure 8, different planes across the nuclei of cells are shown to demonstrate a three-dimensional distribution of GNPs. As-made GNPs did not enter the nucleus because they were trapped in endosomes and lysosomes until excretion (Figure 8A). Only the GNP-BLMs were able to reach the nucleus (Figure 8B). As shown in Figure 9, most of the GNP-BLMs were either closer to the nucleus or were in the nucleus. Our results are consistent with previous studies performed using multifunctional core shell NPs to deliver BLM [2]. Similar observations were made with BLM carrying core shell NPs.

The clinical efficacy of the BLM is thought to stem from their ability to mediate single- and double-strand DNA breaks [49,50]. However, it appears that DSBs are the most lethal ones causing cytotoxicity after interaction of BLM with DNA [38]. Hence, we probed the extent of DNA DSBs as a tool to determine the therapeutic efficacy of GNP-BLMs *vs.* BLM (see Figure 5). It has shown previously that the immobilization of BLM on glass beads via covalent linkage through the C-terminal substituent has little effect on the ability of BLM to cleave DNA [51]. One unrepaired DNB is sufficient to trigger permanent growth arrest and lead to cell death [38]. As illustrated in Figure 5 and Figure 7, Figure 8 and Figure 9, an increase in DNA DSBs can be seen in cells treated with the GNP-BLM complex. Based on our results and previous studies conducted by other research groups, the presence of drug molecules on the surface of GNPs did not hinder the mechanism of action of BLM [51].

The therapeutic effectiveness of BLM is limited due to the side effects [2]. Hence, the use of GNPs generates some answers to the problem of bioavailability. For example, they offer the possibility of transporting major quantities of drugs due to their large surface to volume ratio. In addition, it is possible to capitalize on passive and active targeting of NPs to ensure limited harmful systemic distribution. The enhanced permeation and retention (EPR) effects would facilitate the delivery of drug carrying NPs into solid tumors. Based on the outcome of our study, it is possible to attach the drug onto GNPs without losing its efficacy. Hence, the usage of this particular antitumor drug could be expanded since dosages could be delivered closer to the biological target, such as nucleus and could be contained. Furthermore, the grafting of targeting moieties, such as antibodies, onto NPs allows for active targeting, thus decreasing the interaction with healthy cells. It is also shown that radiation dose enhancement can be improved by targeting GNPs into the nucleus [35]. If we can use BLM conjugated GNPs, it would allow us to capitalize on very effective combined treatment of chemotherapy and radiation therapy to treat most resistant cancer cells [52]. In our study, we were able to reduce the concentration of BLM and GNPs up to nM concentrations for the first time and were still able to see improved therapeutics. Clinically feasible low concentrations of NP-based therapeutics would facilitate the translation of this research into future early phase clinical trials in the near future. The most rewarding factor will be the minimization of side effects of the chemotherapy drugs in order to improve the quality of life of cancer patients.

## 4. Experimental Section

### 4.1. Synthesis of Colloidal Gold Nanoparticles (GNPs)

GNPs of ~10 nm size were synthesized using the citrate reduction method. Firstly, 300 mL of 1% HAuCl_4_·3H_2_O (Sigma-Aldrich, St. Louis, MO, USA) was added to 30 mL of double distilled water and then heated on a hot plate while stirring. Once the solution began boiling, 1000 μL of 1% anhydrous citric acid (Sigma-Aldrich) was added while being stirred. The color of the solution slowly changed from dark purple to bright red. The solution was left to boil for five minutes and was then brought to room temperature while stirring.

### 4.2. Drug Conjugation

GNP-BLM complexes were assembled through sequential conjugation of peptides and BLM onto the GNP surface. A penta-peptide (CALNN peptide) was first added according to the ratio of 300 peptides/GNP for stabilization of GNPs. This is referred to as GNP-PENT in Supplementary Material, Section S1. The RGD-peptide sequence, CKKKKKKGGRGDMFG, was added next at the ratio of 8 peptides/GNP-PENT. BLM was added to the GNP-PENT-RGD complex according to the ratio of 4050 BLM/GNP-PENT-RGD to form the GNP-PENT-RGD-BLM complex. The optimized GNP-PENT-RGD-BLM complex used in this study for drug delivery is referred to as GNP-BLM for simplicity. The medium used was water. For all conjugates, UV-visible spectrophotometry (Shimadzu corporation, Kyoto, Japan), zeta potential measurements, and Dynamic Light Scattering (Malvern Instruments, DLS, Worcestershire, UK) were measured to confirm a minimal shift in size and no aggregation. Additional information is provided in the Supplementary Material, Section S1.

### 4.3. In vitro Experiments with the Drug

MD-MB-231 was cultured in Dulbecco’s Modified Eagle’s Medium (DMEM) supplemented with 10% Fetal Bovine Serum (FBS) at 37 °C in a humidified incubator with 95% air/5% CO_2_. The concentration of GNP-BLM complexes used this study was 1 nM.

### 4.4. Quantification of Nanoparticle Uptake

Following overnight incubation with GNPs, the cells were washed three times with PBS and were trypsinized for quantification of the number of GNPs present per cell. Cells were counted and then treated with aqua regia at 200 °C in an oil bath for ICP-AES analysis.

### 4.5. Clonogenic Assay

After the treatment, the cells were trypsinized and seeded in 60 mm tissue culture dishes. The cells were left in the incubator for two weeks for colonies to form. Methylene blue (0.1%) was used for staining of the colonies. The survival fraction of cells was determined using the ratio of the number of colonies formed/number of cell seeded.

### 4.6. Immunofluorescence Assay

Cells were grown in coverslips (#1.5 18 mm) in 6 well dishes. After the overnight treatment under different experimental conditions (No GNP, GNP, BLM, GNP-BLM), the cells were rinsed three times with PBS. The cells were then treated with 2% paraformaldyhyde/PBS/0.2% and Triton X-100 for 20 min followed by treatment with 0.5% NP40 for 20 min. Cover slips were left in 2% BSA/1% donkey serum in PBS for 1 h. Cells were washed with PBS three times for 5 min between each treatment. Following this, the coverslips were fixed with primary antibodies (53BP1 Ser 1778 and Anti-phospho-histone H2Ax) overnight. The coverslips were then washed with 0.5% BSA/0.175% Tween 20/PBS (secondary wash) for 5 min three times before being treated with optically labeled secondary antibodies (αR Alexa 488 and αM Alexa 647) for 45 min. The coverslips were washed with the secondary wash before being treated with 0.1 μg/mL of DAPI for 10 min. The coverslips were then finally washed with PBS for 5 min three times and mounted onto glass slides after adding a drop of antifade solution. The edges were sealed and stored at 4 °C in the dark.

### 4.7. Apoptotic Assay

The treated cells were washed with PBS and trypsinized. The cells were pelleted in a centrifuge 500xg for 5 min and the supernatant was removed. The cell pellets were re-suspended in the annexin staining buffer. Alexa 647 conjugated annexin V was added for 15–20 min. Propidium iodide (PI) was added to the sample immediately before cytometric measurement.

### 4.8. Hyperspectral Imaging

The CytoViva technology in combination with dark field microscopy was used to image GNP distribution within cells. The illumination of the microscope system utilized oblique angle illumination to create high resolution dark-field images. This imaging system was designed so that in spite of NP interaction with cells or tissue, their spectra may still be confirmed because they are still optically observable. The microscope is a dark-field imaging system that uses oblique angle lighting. NPs appear bright due to high scattering cross-sections of GNPs. To confirm the spectra of GNPs, Spectral Angle Mapping (SAM) was conducted with the CytoViva hyperspectral imaging system (CytoViva Inc., Auburn, AL, USA). SAM determines the presence of GNPs in the input image by comparing unknown spectra in the acquired hyperspectral image to a user-defined spectrum, GNPs in these experiments. This hyperspectral imaging of GNPs in cells and tissues was practical since it does not require optical labeling of the GNPs. It is also possible to extract spectral information from each pixel for verification purposes. More information is provided in the Supplementary Material, Section S1.

## 5. Conclusions

We have succeeded in designing a GNP-based nano-platform (GNP-BLM) to deliver the anticancer drug BLM, while maintaining its cytotoxic activity. Furthermore, GNPs carrying BLM enabled interactions between the BLM and the nucleus, thus resulting in enhanced DNA DSBs. In light of these results, the GNP-based nano-platform proposed here has great potential for the delivery of BLM to tumors while improving its biodistribution.

## Supplementary Materials

The following are available online at http://www.mdpi.com/2079-4991/6/3/48/s1.
