# Supplementary material for: Colloidal Gold-Mediated Delivery of Bleomycin for Improved Outcome in Chemotherapy
Source: Nanomaterials (Basel). 2016 Mar 10;6(3):48. doi: 10.3390/nano6030048 (PMC5302526; doi:10.3390/nano6030048)
Supplement: Supplementary file 1 [file nanomaterials-06-00048-s001.pdf]

Colloidal Gold-Mediated Delivery of Bleomycin for Improved Outcome in Chemotherapy

Celina Yang, Jamie Uertz and Devika B. Chithrani

S1. Characterization and Cellular Uptake of GNPs Functionalized with Peptides and BLM

Gold nanoparticle-bleomycin (GNP-BLM) complexes were assembled through sequential conjugation of peptides and BLM onto GNP surface. Penta-peptide (CALNN) was first added at the ratio of 300 peptide/GNP for stabilization of GNPs and is referred to as GNP-PENT. RGD-peptide (CKKKKKKGGRGDMFG) was added next at the ratio of eight peptide/GNP-PENT followed by BLM at the ratio of 4050 BLM/GNP-PENT-RGD to form GNP-PENT-RGD-BLM complex. The optimized GNP-PENT-RGD-BLM complex used in this study for delivery of BLM in to cancer cells is referred to as GNP-BLM for simplicity. The medium used to conjugate NP-based drug complex was water. The reason is GNP was synthesized using doubled distilled water and peptide molecules and BLM were added sequentially as mentioned before to a vial of GNP solution. The stability of GNP-BLM was monitored for 48 h at 37 °C in a cell incubator. For all conjugates, UV-visible spectrophotometry, zeta potential measurements, and Dynamic Light Scattering (DLS) were measured to confirm the shift in size and no aggregation. In addition, GNP-PENT-BLM was also conjugated (ratio is 1:300:4050) to determine the effect of RGD-peptide in GNP-PENT-RGD-BLM complex. Our focus in this manuscript was GNP-BLM (GNP-PENT-RGD-BLM) *vs.* BLM.

The uptake of GNP-PENT has higher uptake than as-made GNPs. This could be due to the less negative charge of GNPs. Similar results have been demonstrated before by other groups. However, when we added BLM onto GNP-PENT, uptake of GNP-PENT-BLM reduced, as shown in the Figure. However, we don't know the reason for that. It could be due to the interaction of BLM at the cell membrane and also due to intracellular changes as a result of internalization of GNPs with the drug, BLM. This might lead to changes in uptake over a period of time once the drug, BLM, was internalized. However, we were able to increase the uptake of GNP-PENT-BLM by introducing a peptide containing integrin binding domain, RGD (then the complex was referred to as GNP-PENT-RGD-BLM in the Figure). There is ~15% increase in uptake when the GNP complex had RGD-peptide. We saw a similar trend when RGD peptide was incorporated onto GNP-PENT complex.

The NPs were characterized using dynamic light scattering, zeta potential measurements, ultraviolet (UV) visible spectroscopy, and transmission electron microscopy (TEM), as illustrated in Figure S1. We also investigated the variation of uptake as we change the surface properties of GNPs. The increase in uptake of NPs functionalized with penta-peptide alone could be due to their less negative charge. Attaching the RGD peptide containing an integrin binding domain (RGD) onto the complex further improved uptake (*i.e.*, GNP-PENT-RGD). Supplementary Section S4 has shown that RGD peptide can also facilitate their escape from endo-lyso path. This would be necessary if these NPs were to reach the nucleus.

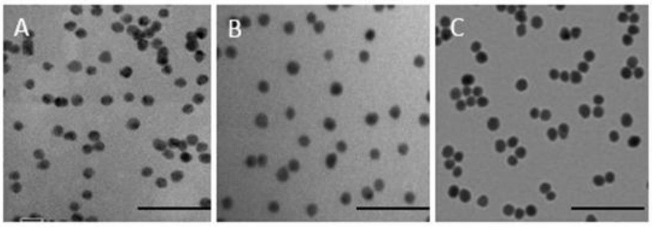

E	Hydrodynamic diameter (nm)	Zeta potential (meV)
GNP	18.8 ± 0.2	-28.23 ± 2.11
GNP-PENT	19.0 ± 0.1	-18.11± 1.12
GNP-PENT-RGD	19.3± 0.2	-12.12± 1.77
GNP-PENT-BLM	19.4± 0.1	-10.91± 1.38
GNP-PENT-RGD-BLM (GNP-BLM)	19.5± 0.1	-7.28± 1.38

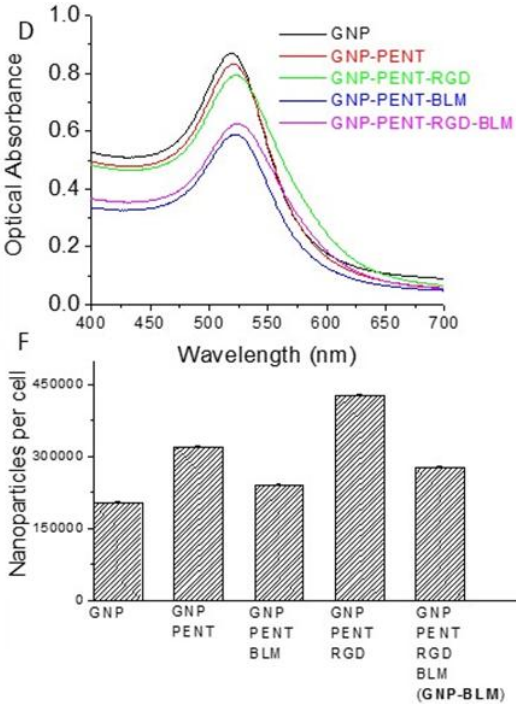

Figure S1. Characterization and cellular uptake of gold nanoparticles (GNPs) functionalized with peptides and BLM. (**A**–**C**) Transmission electron microscopy (TEM) images of as-made GNPs, GNP-PENT-RGD, and GNP-PENT-RGD-BLM, respectively. GNP-PENT-RGD-BLM complex is referred to as GNP-BLM throughout the manuscript; (**D**) Ultraviolet (UV) visible spectroscopy data for as-made GNP, GNP-PENT, GNP-PENT-RGD, GNP-PENT-BLM, and GNP-PENT-RGD-BLM; (**E**) Dynamic light scattering and zeta-potential data for as-made GNP, GNP-PENT, GNP-PENT-RGD, GNP-PENT-BLM, and GNP-PENT-RGD-BLM; (**F**) Cell uptake data for as-made GNP, GNP-PENT, GNP-PENT-RGD, GNP-PENT-BLM, and GNP-PENT-RGD-BLM (or GNP-BLM).

S2. Hyperspectral Imaging of GNPs Localized within Cells

We used hyperspectral imaging (HIS) technology to image GNPs. This technology allows us to image NPs without having to labeling them with fluorescent molecules. This technique use high scattering power of GNPs to generate the image. GNPs localized within the cell can be mapped using a reference reflectance spectra (white color spectrum in Figure S2.1A) corresponding to GNPs. Figure S2.1B is a cross section of an unstained cell and has internalized GNPs. GNPs localized with the cell appears as white bright got like structures. The red dots are the mapped GNPs according to the reference spectra shown in Figure S2.1A. As illustrated in this Figure S2.1B, no GNPs were localized within the nucleus since citrate capped GNPs cannot enter the nucleus. Most of the NPs were localized within the cytoplasm and we can see many bright dots. The spectrum taken from these bright dots represent reflectance spectra of GNPs. Figure S2C shows reflectance spectra from many bright dots shown in Figure S2.1A. We have also taken background spectra from nucleus (Figure S2.1B; green spectrum) and cytoplasm (Figure S2.1B; red spectrum). The spectra from nucleus and the cytoplasm were almost flat. Hence, using this technique, we can image the NPs (with unique reflectance spectrum) localized within the cell. In the next section, we will show how to differentiate GNPs localized within the cytoplasmic vesicles and nucleus.

GNPs localized within the nucleus *vs.* cytoplasm are shown in Figure S2.2. The area of nucleus is enclosed using a yellow dotted line for clarity. According to Figure S2.2A, GNPs were localized only within the cytoplasm, but not in the nucleus. The reflectance spectra taken from few GNP clusters (marked with red dots) are shown in Figure S2.2B. According to Figure S2.2C, GNPs were localized within both cytoplasm and nucleus. The reflectance spectra taken from few GNP clusters (marked with red dots) are shown in Figure S2.2D.

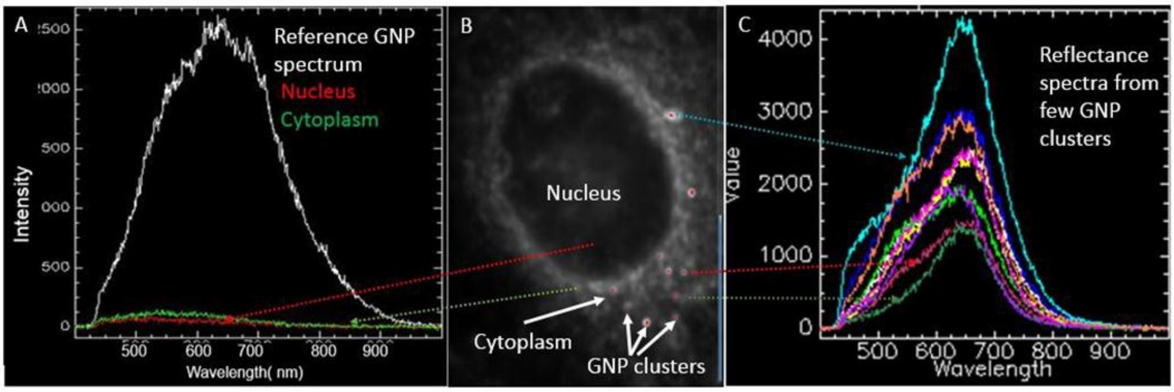

Figure S2.1. Hyperspectral imaging of cells with localized GNPs. (**A**) Reference spectra corresponding to GNPs, nucleus, and cell cytoplasm; (**B**) Dark field image of a cell cross section showing localized GNPs as white bright dots. The small red dots on top of white bright dots represent the GNPs mapped according to the reference spectrum of GNPs; (**C**) Individual reflectance spectra of few selected GNP clusters localized within the cell shown in (**B**).

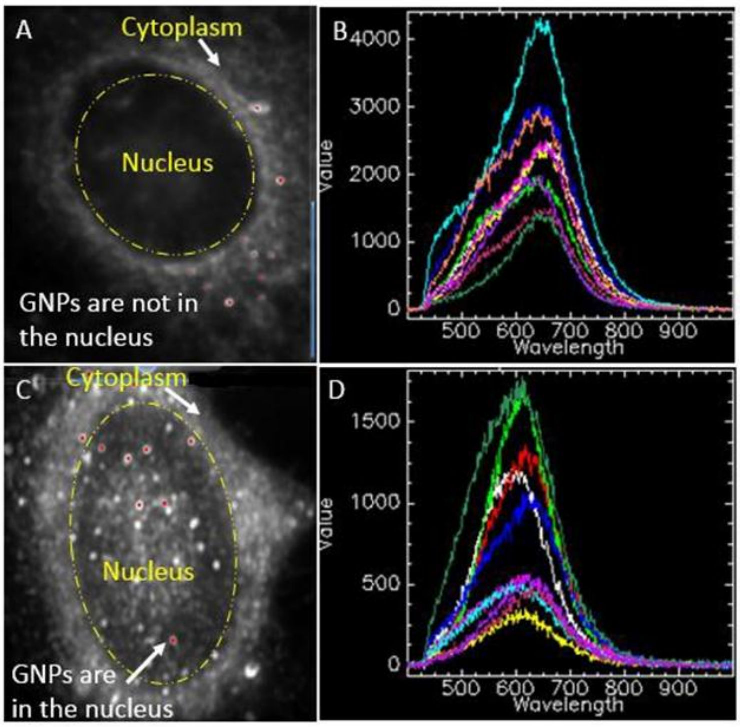

Figure S2.2. Cross-section through the nucleus of cells to show GNPs localized in cytoplasmic vesicles and nucleus (enclosed with the yellow dotted line). (**A**) Dark field image of a cell where GNPs are localized in the cytoplasmic vesicles (small white color bright spots). No GNPs are localized within the nucleus; (**B**) Reflectance spectra of few GNP clusters (labeled as red dots) localized in the cell shown in (**A**); (**C**) Dark field image of a cell where GNPs are localized within the nucleus and cytoplasm (small white color bright spots). GNPs are localized within the nucleus; (**D**) Reflectance spectra of few GNP clusters (labeled as red dots) localized in the cell shown in (**C**).

S3. The Viability of the Cells at the Concentration of GNPs Used in this Study

The viability of the cells in the presence of as-made (or citrate capped) GNPs *vs.* control (cell with no internalized GNPs) was tested using clonogenic cell survival assay and DNA DSBs assay, as illustrated in the figure below. The presence of GNPs did not introduce any significant toxicity at the concentration (1 nM) used for this study

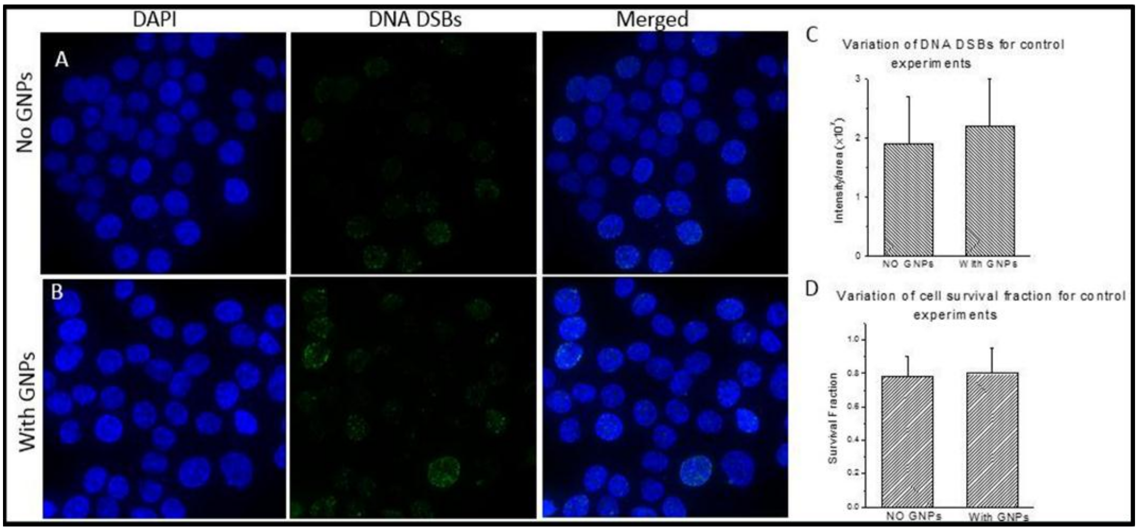

Figure S3. Viability of cell with and without GNPs. (**A**–**B**) Optical images showing that control cells with no GNPs and cells treated with as-made (or citrate capped) GNPs (**B**) did not show any significant difference in DNA double strand breaks (DSBs). (**C**–**D**) Quantitative data show that as-made GNPs did not compromise the viability of the cells at the concentration used for this study. DNA DSBs assay (**C**) and cell survival assay (**D**) showed no significant change in cell viability in the presence of as-made GNPs.

S4. The Role of RGD-Peptide

Our TEM data showed that as-made GNPs were always trapped in cytoplasmic vesicles (Figure S4A1, S4A2) until they get excreted from the cell while GNPs functionalized with RGD-peptide could escape the cytoplasmic vesicles and localized in the cytoplasm (Figure S4B1, S4B2, S4C1, and S4C2).

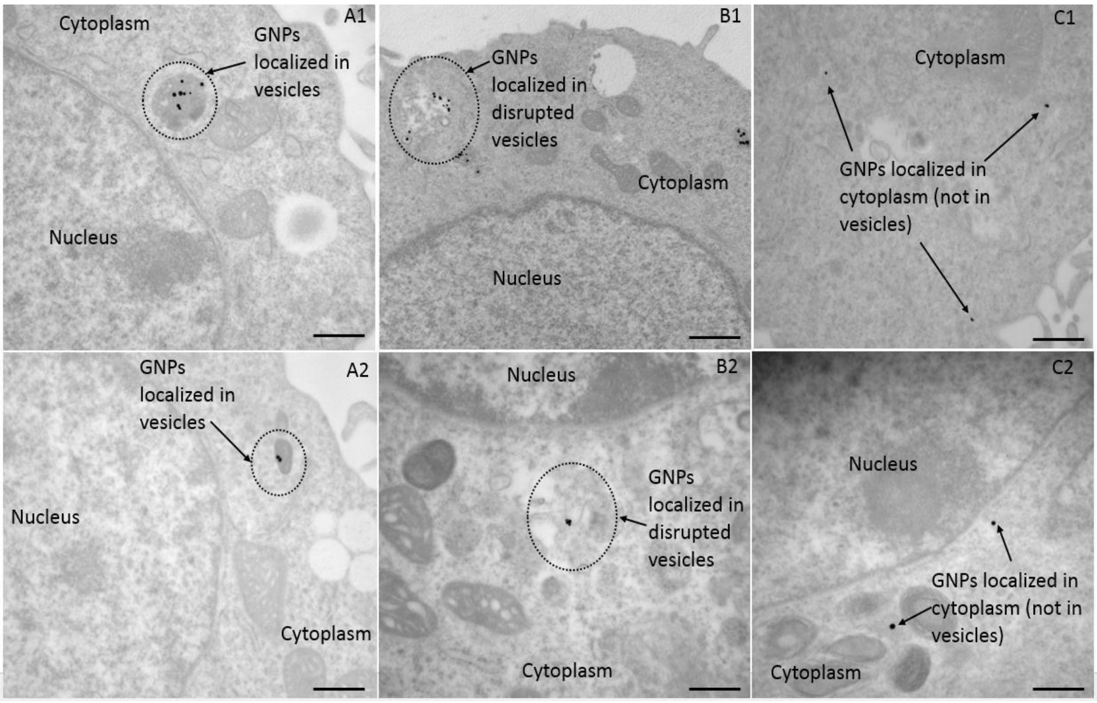

Figure S4. TEM images showing distribution of GNPs within cells based on their surface functionality. (**A1**,**A2**) TEM image cross section of a cell showing the as made GNPs were localized in small vesicles (endosomes or lysosomes) localized in the cytoplasm. (**B1**,**B2**) GNPs functionalized with penta-peptide and RGD peptide can escape these vesicles by disrupting the membrane. (**C1**,**C2**)

GNPs functionalized with penta-peptide and RGD peptide can localize in the cytoplasm by themselves after escaping the cytoplasmic vesicles as shown in B.

S5. Viability of Cells Treated with no GNPs and as-Made GNPs *vs.* Cells Treated with BLM and GNP-BLM

We have investigated the variation of cell survival fraction and DNA DSBs for cells internalized with no GNPs (control), as-made GNPs, BLM, and GNP-BLMs. This Figure clearly shows that conjugation of BLM to GNPs can increase the therapeutic efficacy *vs*. BLM alone, while as-made GNPs did not cause significant toxicity at the concentration (1 nM) used for the study.

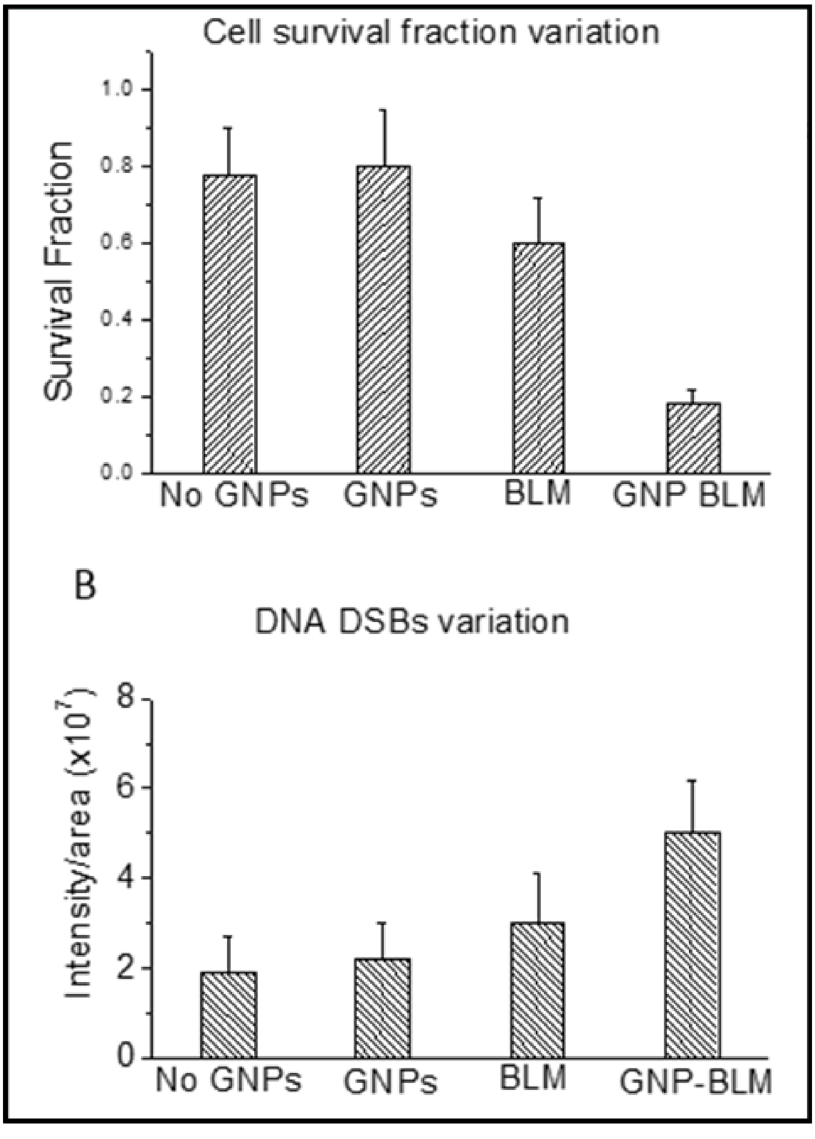

Figure S5. Determination of cell survival fraction (**A**) and DNA DSBs (**B**) for GNP-BLM *vs.* BLM, as-made GNPs, and control (No GNPs) (n = 3).

S6. Viability of Cells Treated with no GNPs and as-Made GNPs *vs.* Cells Treated with BLM and GNP-BLM

Results of DNA DSBs assay for cells treated with no GNPs (control), as-made GNPs, BLM, and GNP-BLMs. More DNA DSBs are seen for cells treated with GNP-BLMs *vs.* BLM. This Figure clearly shows that conjugation of BLM to GNPs can increase the therapeutic efficacy *vs.* BLM alone, while as-made GNPs did not cause significant toxicity at the concentration (1 nM) used for the study.

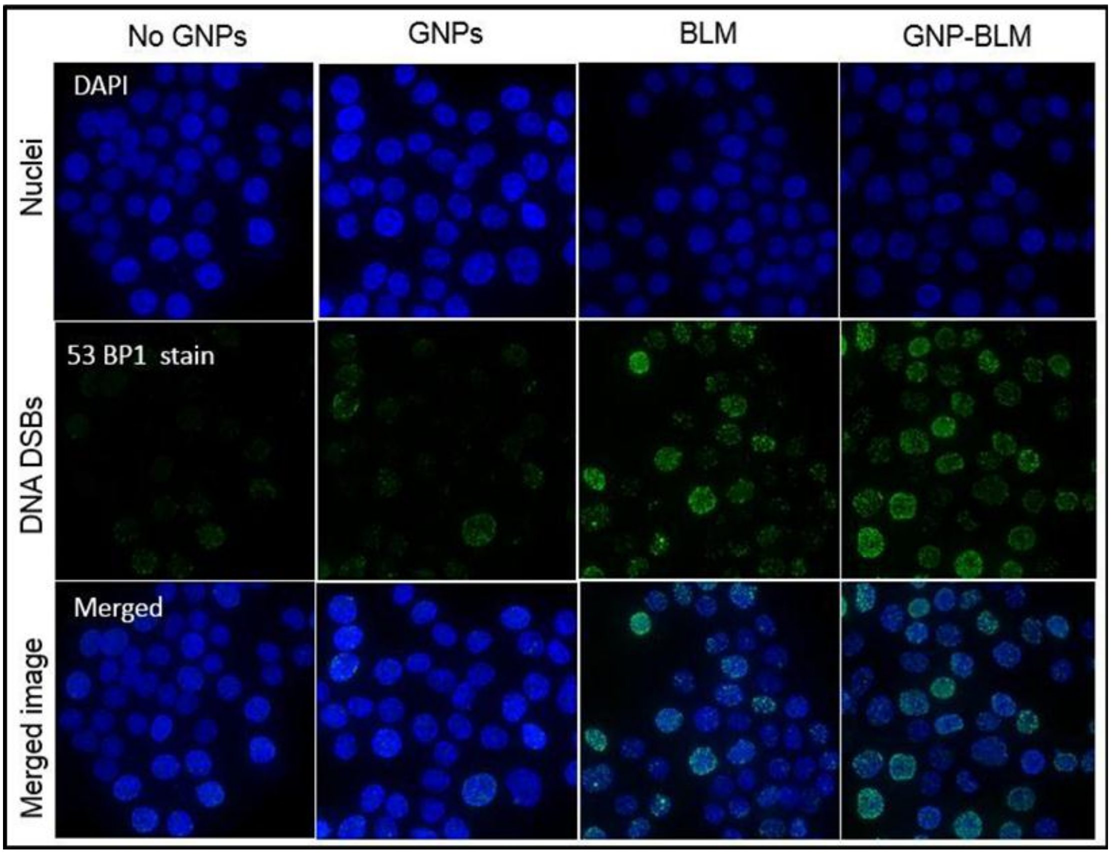

Figure S6. Measuring the efficacy of the drug, BLM, using DNA DSBs assay. Optical images of DNA DSBs in MDA-MB-231 cells incubated with no GNPs (control), as-made GNP, BLM alone, and GNP-BLM after 24 h of the treatments. The first represent nuclei stained with 4',6-diamidino-2-phenylindole (DAPI), the second row represents DNA DSBs probed with optically tagged antibodies, and the third row represent overlay of images.

© 2016 by the authors; licensee MDPI, Basel, Switzerland. This article is an open access article distributed under the terms and conditions of the Creative Commons by Attribution (CC-BY) license (http://creativecommons.org/licenses/by/4.0/).
